# Current Implementation Outcomes of Digital Surgical Simulation in Low- and Middle-Income Countries: Scoping Review

**DOI:** 10.2196/23287

**Published:** 2023-06-15

**Authors:** Arnav Mahajan, Austin Hawkins

**Affiliations:** 1 Department of Medicine University College Cork Cork City Ireland

**Keywords:** adaptation, digital surgery, global surgery, simulation, surgery, systematic review, technology, video game

## Abstract

**Background:**

Digital surgical simulation and telecommunication provides an attractive option for improving surgical skills, widening access to training, and improving patient outcomes; however, it is unclear whether sufficient simulations and telecommunications are accessible, effective, or feasible in low- and middle-income countries (LMICs).

**Objective:**

This study aims to determine which types of surgical simulation tools have been most widely used in LMICs, how surgical simulation technology is being implemented, and what the outcomes of these efforts have been. We also offer recommendations for the future development of digital surgical simulation implementation in LMICs.

**Methods:**

We searched PubMed, MEDLINE, Embase, Web of Science, Cochrane Database of Systematic Reviews, and the Central Register of Controlled Trials to look for qualitative studies in published literature discussing implementation and outcomes of surgical simulation training in LMICs. Eligible papers involved surgical trainees or practitioners who were based in LMICs. Papers that include allied health care professionals involved in task sharing were excluded. We focused specifically on digital surgical innovations and excluded flipped classroom models and 3D models. Implementation outcome had to be reported according to Proctor’s taxonomy.

**Results:**

This scoping review examined the outcomes of digital surgical simulation implementation in LMICs for 7 papers. The majority of participants were medical students and residents who were identified as male. Participants rated surgical simulators and telecommunications devices highly for acceptability and usefulness, and they believed that the simulators increased their anatomical and procedural knowledge. However, limitations such as image distortion, excessive light exposure, and video stream latency were frequently reported. Depending on the product, the implementation cost varied between US $25 and US $6990. Penetration and sustainability are understudied implementation outcomes, as all papers lacked long-term monitoring of the digital surgical simulations. Most authors are from high-income countries, suggesting that innovations are being proposed without a clear understanding of how they can be incorporated into surgeons’ practical training. Overall, the study indicates that digital surgical simulation is a promising tool for medical education in LMICs; however, additional research is required to address some of the limitations in order to achieve successful implementation, unless scaling efforts prove futile.

**Conclusions:**

This study indicates that digital surgical simulation is a promising tool for medical education in LMICs, but further research is necessary to address some of the limitations and ensure successful implementation. We urge more consistent reporting and understanding of implementation of science approaches in the development of digital surgical tools, as this is the critical factor that will determine whether we are able to meet the 2030 goals for surgical training in LMICs. Sustainability of implemented digital surgical tools is a pain point that must be focused on if we are to deliver digital surgical simulation tools to the populations that demand them the most.

## Introduction

### Background

Safe surgical care is an often-neglected component of health systems, with an estimated 5 billion people lacking access [1]. According to The Lancet Commission for Global Surgery, only 6% of surgeries are performed in the poorest countries, despite the fact that they contain one-third of the world’s population. Education and training of the workforce was identified as a crucial issue, with massive shortages of certified surgeons constituting a significant barrier to care in low- and middle-income countries (LMICs). To address the care shortage, it was suggested that the surgical, anesthetic, and obstetric workforce in LMICs be increased to 40 per 100,000 population by 2030 [1]. Despite the fact that traditional models of surgical training adopted in high-income countries (HICs) include a system of graded autonomy that spans up to 7 years of training, up to 30% of these trainees do not feel confident operating independently after residency [2,3]. Given the constraints imposed on surgical education in many LMICs, this failure to cope with a large surgical disease burden is directly responsible for worse patient outcomes [4].

These factors have effects that extend beyond the operating room and have led to a large brain drain of skilled trainees to other countries in search of more material resources to pursue robust surgical training [5]. This is exacerbated by the difficulty trainees face in accessing relevant literature translated into their language that is context specific to the unique and complex disease presentation in LMICs [6]. Existing solutions to combat this have been proposed, such as development of surgical simulation suites, but these require a significant amount of resources; increasing access to cadaveric and animal model simulations, but this requires additional training and specialized staff; and low-fidelity simulation, but this lacks the sophistication of the advanced techniques used in this field that evolve into more refined approaches of care [7-10]. Innovative simulation-based tools, such as virtual reality (VR), augmented reality (AR), and tele-simulation applications, are best suited for trainees who want to improve their skills in light of the aforementioned obstacles [11,12]. We use the digital domains of digital surgery, previously defined in detail within the HIC literature, to define the scope of this study and the investigated term, *digital surgical simulation*, including smartphone apps, sensors, VR, AR, artificial intelligence, and robotics [13]. In HICs, these technologies have been used to improve surgical performance and patient safety; however, the impact of these technologies in LMICs is unknown.

Despite the shift in surgical training methodology, studies qualifying the efficacy of digital surgical training in LMIC settings are lacking. Although it has been demonstrated that surgical simulation is a highly effective way to scale up training in HICs, the implementation barriers within LMICs are unknown [9,11,12]. Understanding clinical outcome and benefit is essential, but if the outcomes cannot be implemented in practice, the technology remains ineffective and only useful in theory. Therefore, it is crucial to study the implementation of these technologies. With the urgent need to scale up training in LMICs, our global innovation efforts may be ineffective if we do not assess implementation in this context.

In light of this, we intend to investigate the implementation outcomes of digital surgical simulation tools in LMICs by conducting a scoping review. Given the heterogeneous literature examining a variety of tools, surgical procedures, and LMICs with distinct and context-specific problems, a scoping review is the most appropriate method for answering this question.

### Objectives

In this study, we will conduct a scoping review of all the current surgical trainees and practitioners in LMICs who use digital surgical simulation tools, and we will conceptualize these findings using the implementation outcome framework. Our objectives will be to determine which types of surgical simulation tools have been most widely used in LMICs, how surgical simulation technology is being implemented, and what the outcomes of these efforts have been. We also offer recommendations for the future development of digital surgical simulation implementation in LMICs.

## Methods

### Overview

This scoping review was conducted in accordance with the Joanna Briggs Institute (JBI) methodology [14]. Full search results were reported and displayed in a Preferred Reporting Items for Systematic Reviews and Meta-Analyses Extension for Scoping Review (PRISMA-ScR) flowchart [15]. In addition, we have completed a PRISMA-ScR checklist (Multimedia Appendix 1 [15]). A preliminary search of MEDLINE, Cochrane, PubMed, and PROSPERO did not reveal any active or forthcoming reviews on this subject.

### Search Strategy

For this study, PubMed, MEDLINE, CINAHL, Web of Science, Embase, and the Central Register of Controlled Trials were searched. Before title screening, abstract screening, and full text review in Rayyan, the results were exported to EndNote (version X8; Clarivate) to remove duplicates. No limitations were placed on the original publication language or date (last search was completed on March 12, 2022). Any papers that were not written in English were translated using Google Translate (Alphabet Inc) to account for the literature published specifically for LMICs that was written in a specific language. The search string was generated by searching sources and developing pertinent search terms that were tested for sensitivity in advance of this review by a previous analysis of PROSPERO study protocols and key term analysis of the literature. For this search, we used the World Bank’s definitions of LMICs, Atallah’s [13] framework for defining the scope of digital surgery and near-terms, and Proctor et al’s [16] classification of implementation outcomes. We chose to remain rigid to these terms as the scope of this paper is to examine how well these tools have been implemented, not whether the tools exist or not, as the implementation of these tools is arguably a more important factor in determining their success and reproducibility (Textbox 1).

Guidelines for reporting conformed to PRISMA Scoping Review requirements. These terms have been modified to search the specifics of each database and are accessible (Multimedia Appendix 2).

**Eligibility criteria.**

**Study types**

Given the nature of this paper to study implementation outcomes, for a study to be eligible for inclusion the paper must describe and report outcomes on the specific effectiveness of a given intervention through explicit testing of implementation strategy. As such, they must fall within the “effectiveness-implementation” hybrid model first described by Curran et al [17]. Excluded papers were secondary studies such as systematic reviews and nonempirical studies such as books, protocol, viewpoints, and commentaries.

**Participants or population**

Study participants from LMICs were included, according to the World Bank definition, who were surgical trainees or practitioners at any level of their training. Surgical obstetric care was included as a part of this review. Excluded participants were those who were not medical degree holders but are allied health care professionals that engage in task-sharing—a novel practice being introduced into LMICs to address the human resource gap [18].

**Intervention or exposure**

This review will focus on studies that have implemented or evaluated a digital surgical simulation tool. We have defined digital surgical simulators as innovations that allow trainees to develop surgical skills through use of digital technology by a hands-on approach based on previously published literature [13]. These may include virtual reality, augmented reality, serious games, tele-simulations, tele-proctoring. Patient-specific anatomy that has been rendered into a virtual reality model utilizing 3D modeling was included. Studies were excluded if the digital surgical simulator described was a web-based or flipped classroom model. Similarly, studies that used 3D-printed models as simulators were excluded as these are not digital simulations.

**Control**

Eligible studies will compare implementation interventions (digital surgical simulators) in terms of effectiveness by looking at surgical competency before and after use of the simulator. Studies may also compare participants' baseline confidence in conducting the surgery. Studies that compared control intervention (conventional simulation, animal and cadaveric simulation, or lecture-based education models) were also included.

**Outcome**

As a part of this study, implementation and quantitative evaluation of the digital surgical simulators for surgical trainees must be included. At least one outcome measure must be reported to be included as a study. We use Proctor et al’s [16] study to describe the specific 8 sub-classifications of implementation success of digital surgical simulators of acceptability, adoption, appropriateness, feasibility, fidelity, implementation cost, penetration, and sustainability. This model has historically been used in a Wellcome study protocol [19] to explore low-technology simulation for training in LMICs for surgical intervention of gastroschisis and as such is relevant for this study as well.

### Data Synthesis and Extraction

Following the search, all citations were compiled and uploaded to EndNote X8 for duplicate removal. Two reviewers carried out a title and abstract screening (AM and AH). The references of included articles were examined to determine whether additional literature should be included. Using the inclusion criteria, the full texts of selected papers were carefully evaluated. In the scoping review flowchart, the reasons for excluding full-text evidence sources that did not meet the inclusion criteria were recorded and reported.

Using a data extraction tool adapted from the JBI methodological template and supplemented with framework items from Proctor et al [16], we extracted data from the papers included in the scoping review [14]. The extracted data will include specific information regarding the study’s location, objectives, study design, type of digital surgical simulation, number of individuals trained, acceptability, adoption, adequacy, feasibility, fidelity, implementation cost, penetration, and sustainability, as well as key findings pertinent to the review questions. This approach to data extraction is comparable to previously published methods [20]. Described study characteristics were followed by a summary of results based on Proctor et al’s [16] subclassification. If there is insufficient information on a particular subclassification, these taxonomy components were removed from qualitative analysis and an appropriate explanation was provided. Due to the heterogeneity of this paper’s scope, quantitative analysis between papers was omitted in favor of qualitative and narrative descriptions of included papers in order to answer the research question and achieve the objectives. The extraction sheet with specifics is available in Multimedia Appendix 2.

We determined the suitability of instruments using Proctor et al’s [16] concept of implementation outcomes, despite the fact that the constructs did not always fit neatly within the established objectives. Where the description of such constructs fit more than one of Proctor et al’s [16] outcomes, the instrument was categorized according to the outcome that predominated, as determined by a comprehensive study and count of every instrument item. In the absence of a clear distinction, taxonomy components were thematically grouped and analyzed qualitatively. If tools evaluated additional components outside of the taxonomy, we did not include them in our extraction of the articles; however, we did analyze thematic parallels between the reporting in this paper. Any disagreements that arise between the reviewers are resolved through discussion, if applicable.

Given the heterogeneity of indications and outcomes of digital surgical simulation for trainees in LMICs, no meta-analysis was conducted. Instead, a mixed-methods analysis of the extracted literature was conducted in consideration of our implementation outcome model. The individual sources of evidence were not evaluated in accordance with JBI protocol.

Our search method restricted the discovered publications to the implementation of Proctor et al’s [16] taxonomy results. This may result in the removal of pertinent publications that examined digital surgical instruments in LMICs. However, given that researchers have previously relied on Proctor et al’s [16] framework due to the pragmatic nature of its content in the broader surgical simulation literature, we determined that Proctor et al’s [16] framework is the most relevant context- and intervention-specific framework to evaluate digital surgical tools in LMICs [19].

## Results

### Included Studies

**Figure 1:**
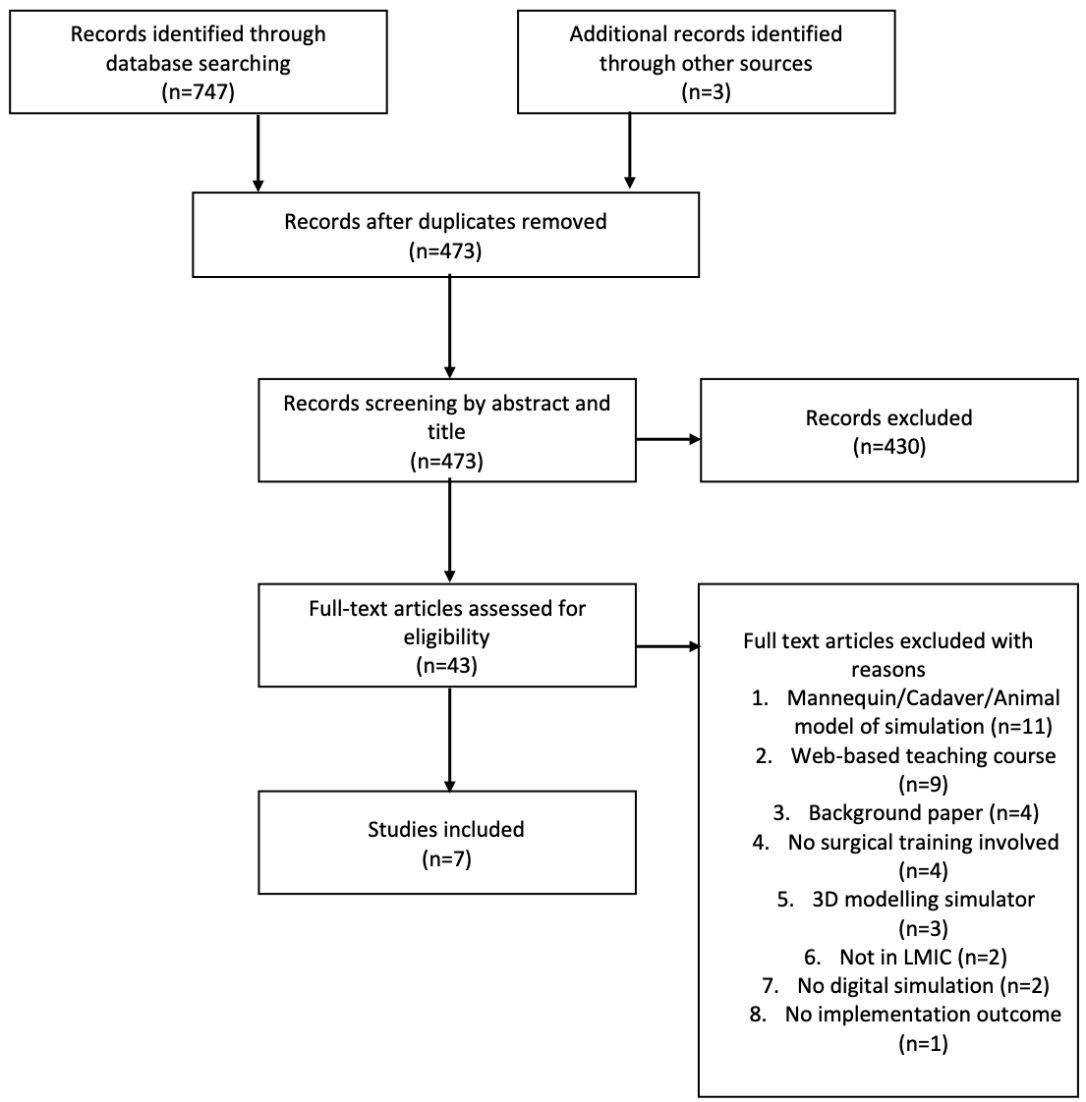
Preferred Reporting Items for Systematic reviews and Meta-Analyses Extension for Scoping Review (PRISMA-ScR) checklist. LMIC: low- and middle-income country.

The database search revealed 747 papers with an additional 3 added from other sources through scanning of bibliographies of papers. After sorting of duplicate papers, 473 papers were included. These were subsequently screened and searched according to title and abstract screening and 43 papers were left. Seven papers remained after excluding other papers by full text. Reasons are listed in Figure 1.

### Study Characteristics

Among a total of 7 studies, there were 3 cross-sectional observational studies, 2 case studies, and 2 randomized controlled trials [20-26]. In 74% (81/110) cases, medical students and residents were the intervention group. A total of 72% (79/110) of the participants in the studied cohorts were men, compared to 28% (31/110) women. The included studies were conducted in 9 different nations. The majority of authors were from the United States, with 6 of the 7 first authors hailing from HICs. Participants number ranged from 2 to 30. The most used digital surgical tool was a VR-based model, used in 4 studies, which was considered to be cost-effective and of low fidelity, or cost-prohibitive with higher fidelity. This was followed by tele-proctoring tools in 2 studies and app-based training tools in 1 study. Indications for design varied with tools being developed across the spectrum of surgical burden including orthopedic, vascular, obstetric, and minimally invasive surgeries. The evaluation of the evidence was not performed in accordance with the JBI’s recommendations.

### Synthesis of Proctor’s Classification

#### Acceptability and Adoption

A review of 4 studies highlighted the acceptability and implementation outcomes of surgical simulators and telecommunications devices in LMICs [21-24]. All participants rated the acceptability of the 3D VR gesture-mediated simulator as attractive. One hundred percent of those polled believed that the prototype could be a solution for ubiquitous learning in minimally invasive surgery [21]. In addition, in the VR simulator of an open radical abdominal hysterectomy, participants reported that the simulation they experienced was similar to their university hospital’s operating room as a digital replica of the theater’s equipment, instruments, supplies, and lighting [22]. Surgical students who used VR as a learning and practice tool for lower limb amputation reported significantly higher levels of engagement in their course. The same students who used VR to study reported higher levels of perceived learning [23]. Students who used a virtual surgery app to prepare for tendon repair simulation rated it as a useful or very useful training and assessment tool 92% of the time, and as a useful or very useful rehearsal tool 85% of the time. Note that 62% of these students indicated that it would be a good or very good curriculum requirement [24].

#### Appropriateness, Feasibility, and Fidelity

Six reviewed studies addressed the appropriateness, feasibility, and fidelity of surgical simulator and telecommunication device implementation outcomes in LMICs [21-26]. On a Likert scale, 94% of students who used a 3D VR gesture-mediated simulator for training rated the tool highly for appropriateness. A total of 93% of participants rated the ability to realistically represent and test hand-eye coordination, and 87% rated the ability to realistically represent depth perception. All of the participants highly rated the device’s usability; however, they commented on how physical forces represented in the virtual environment were less than ideal. There was no significant difference between the expert (practicing surgeon) and referent (surgical residents) groups in any of these fidelity scores [21]. The participants interviewed who used the VR simulator for an open hysterectomy reported that the simulator increased their anatomical and procedural knowledge. In addition, they believed that the skills acquired in the simulator could be applied to other aspects of medical care and practice. The simulator, according to students and surgeons who used it, bolstered their anatomical knowledge and helped them manage complications in the operating room [22]. Surgical residents who received VR instructions on lower limb amputations earned higher scores on average, but the SD overlapped [23]. Surgical residents whose operative skills in a tendon repair simulation were graded by raters demonstrated a disparity between how they prepared for the test and how their skills were evaluated. Touch Surgery, the virtual phone app, resulted in a mean rubric score of 89.71% for students, while textbook learning resulted in a mean score of 63.4% (*P*<.001) [24]. The 2 surgeons who used Google Glass to coordinate field operations in Mozambique reported that the technology was extremely useful as an intraoperative and perioperative training tool. Nevertheless, both participants reported moderate visual impairment due to image distortion and excessive light exposure. Additionally, video stream latency and connection interruptions were cited as limitations [25,26]. Surgeons in Ecuador who were tele-mentored by a Yale University surgeon found their mobile-based, low-bandwidth telemedicine app to be effective in supporting remote health care delivery [26].

#### Implementation Cost

A total of 4 studies reported the cost to implement their unit [21,22,25,27], while the remaining studies [23,24,26] listed their equipment so that the reader can infer the cost to implement. The creators of the 3D VR gesture-mediated simulator for learning fundamental psychomotor skills in minimally invasive surgery spent a total of US $200, excluding software costs, to build their device [21]. Without software licenses, the low-cost VR open hysterectomy simulation setup using an Oculus Rift (Meta Platforms) headset and hand controllers was estimated to cost slightly less than US $1500 [22,27]. The Google Glass device telecollaboration setup used by the 2 surgeons in Mozambique and the United States cost US $999 for the Google Glass device and a yearly subscription fee of US $6990 for the required AMA XpertEye software (Tracxn Technologies). In addition, one required 2 computers or laptops and a Wi-Fi connection [25]. The remaining studies listed required products without associated costs. The Lower Limb Surgical Amputation Virtual Reality Tutorial Study used an unspecified Oculus VR headset [23]. According to the tendon repair study, the Touch Surgery smartphone app costs US $25 [24]. The only requirements for the design of the telecommunications study conducted in Ecuador were an internet connection, 2 laptops with a single camera, and telemedicine and video conferencing software [26].

### Penetration and Sustainability

Penetration and sustainability of digital surgical simulation were heavily underreported outcomes. Both refer to project implementation over a longer scale; penetration refers to the degree to which a new technology has been adopted and used, and sustainability refers to the long-term viability of a technology within their specific contexts. No paper provided results on either of these outcomes, however reference was made to hypotheses from authors of how participants could be willing to incorporate digital surgical simulation into regular training regimens. Additionally, sustainability was neglected in reporting with no reference made to sustainability in terms of cost, upkeep, widespread adoption among all trainees, and implementation on a wider scale.

## Discussion

### Overview

The scoping review examined the outcomes of digital surgical simulation implementation in LMICs. The majority of participants were medical students and residents who were identified as male. Participants rated surgical simulators and telecommunications devices highly for acceptability and usefulness, and they believed the simulators increased their anatomical and procedural knowledge. However, limitations such as image distortion, excessive light exposure, and video stream latency were frequently reported. Depending on the product, the implementation cost varied between US $25 and US $6,990. Penetration and sustainability are understudied implementation outcomes, as all papers lacked long-term monitoring of the digital surgical simulations. The fact that the majority of authors are from HICs suggests that innovations are being proposed without a clear understanding of how they can be incorporated into surgeons’ practical training. Overall, the study indicates that digital surgical simulation is a promising tool for medical education in LMICs; however, additional research is required to address some of the limitations in order to achieve successful implementation, unless scaling efforts prove futile.

### Results in Context

Our findings must be contextualized within the larger body of literature. Although our findings indicate that training using digital surgical simulation may be effective, the Lancet Commission reports that all digital surgical tools should be used as a supplementary resource and not as a primary resource, which would drain hospital resources and compromise patient safety [1]. Several factors are implicated in the context of Proctor et al’s [16] taxonomy. To begin with, it appears that little emphasis is placed on understanding what the implementation costs are. Rather, many authors hope that the tool’s novelty will be sufficient to ensure its successful implementation. If we are to scale technologies across the regions that have the greatest demand for them, implementation must be incorporated with greater consideration. One strategy revealed that participants placed a great deal of emphasis on mentoring, suggesting that mentor-champions must be assigned to medical students and surgical trainees to encourage implementation of these technologies in order to scale use in their respective environments [22]. Moreover, while technically all of these tools may be feasible, the implementation of these tools in contexts that none of the HIC lead authors may be aware of is of greater importance [26]. This was countered by a single study that attempted to replicate the exact visual field of the operating room [22]. However, more thoughtful integration of LMIC authors and incorporation of specific implementation strategies is urgently required.

Cost of development is an important factor to consider when evaluating the eventual uptake of digital surgical simulators in LMICs. When learning how to use a 3D, VR-generated simulator for psychomotor skills in surgeons, one such device costs US $200. However, this did not include software costs or the possibility of recurring fees for subscription-based models. It is crucial to recognize that in the delivery of educational content, the requirement to register for software is a direct barrier to long-term content access. It has been made abundantly clear the significance of developing technology that is easily consumable offline and relevant to local clinical practice [28]. The ideal situation would be one that does not require continuous mobile phone data as well, since limitations of continuous and reliable internet access are still prevalent despite the increasing use of smartphones in professional settings. Using Oculus Rift headsets, a commercial brand with a proven track record of quality and dependability, the costs are approximately US $1500, with software licensing not being recognized in the literature as a recurring cost that could negatively impact the future sustainability of many of these surgical simulators. Although these may appear to be high costs, it is important to note that they are significantly less than those of many surgical mission trips. In addition, some innovations only required a camera and an internet connection, which eliminates travel expenses entirely [26]. Extremely low-cost VR and AR technology is being developed for use with smartphone apps and low-cost headsets, such as Google Cardboard, to use immersive technologies—with the clear recognition that wearable immersive technologies have contributed to a sustainable model of training in low-resource settings [29].

Acceptability was frequently rated quite highly across the majority of studies and was reported by the vast majority of reviewed studies. Responses indicated that the reality of the surgery and the virtual simulation were consistent. This is often in stark contrast to traditional methods of simulation, which lack an understanding of unique and complex 3D structures and fail to improve our understanding of how instruments are handled in the operating room [30]. In orthopedic settings in countries with a high standard of living, the development of curricula with training modules for digital surgical systems demonstrates encouraging results [31]. Novel alternatives, such as printing low-cost 3D silicone models for perineal repair and simulating cricothyroidotomy, have been demonstrated in the literature [32,33]. Depending on the indication, however, these models may be of high fidelity or low fidelity. In silicone models, the lack of simulated fascia, fat, and tissue reduces the responsiveness of the absence of haptic and tactile feedback observed in digital surgical simulations. In addition, although these models may be less expensive, they may not function as intended, with some requiring frequent updates and modifications to a multitude of models that already take up to 11 hours to print [32]. In a study published with the help of the College of Surgeons of East, Central, and Southern Africa (COSECSA), 3D models were cited as the most preferred tool for surgical simulation (45%), with slightly more than 30% of participants seeking VR-based simulations [34]. Approximately 35% of participants found low-cost training models to be the least preferred option. The path forward for surgical trainees in LMICs appears to be paved with innovation and unique simulation techniques. Traditional methods such as animal and cadaver dissection are being phased out of medical school education, despite their undeniable utility. By acknowledging these structural obstacles, acceptance and adoption of novel technologies are increasing. It is essential for trainees in LMICs to be able to engage in simulation without leaving the workplace, as this would ultimately increase the surgical simulator’s acceptance and usage.

When evaluating the success of the implementation of a novel technology, sustainability is a crucial factor to consider. It has been demonstrated that for 67% of all COSECSA trainees, learning surgical techniques with new technology was the most beneficial method of education [34]. However, 85% of the time, a lack of suitable tools and models was cited as a barrier to successful implementation, and 49% of the time, maintenance of facilities for residents was cited as a barrier. Interestingly, since the majority of trainees experienced simulation teaching as a short crash-course model of instruction with little long-term follow up and poor engagement that they could continuously act upon in their own time, this may be the preferred model of instruction for many. In one of the studies we observed, participants assigned to the intervention group continued to use it throughout the duration of the study, demonstrating that the authors recognized the benefit it provided the participants and gave them the opportunity to use such a novel technology [22]. In the broader literature, long-term studies of implementation have been demonstrated with collaborations lasting up to 30 years. Taking into account the challenges of educating and training skilled surgeons, it is possible to study how sustainable these new training models will be in practice [29]. To add to this point about sustainability, fidelity of the instruments and their adaptability to an ever-evolving world of surgical advancement are required. Through their inherent ability to update and modify over time, digital surgical simulators may be able to circumvent this obstacle and reduce implementation costs while extending the device’s sustainability. It is interesting to note, however, that although the fidelity of each simulator may seem important at first glance, it has been demonstrated that the use of high-fidelity simulation models is not significantly superior to the use of low fidelity simulation models. Consequently, in areas with limited resources, low fidelity simulation models may be used [35].

### Limitations

The limitations of this paper are as follows. First, as previously discussed in the methods section, our search strategy may have screened out papers based on our search string criterion; however, we chose to adhere to this as it has been previously outlined in extant global digital surgical literature that examines implementation outcomes in LMICs that such an approach is appropriate. Second, there was a high degree of inconsistency and vagueness in the reporting of implementation outcomes. Although the purpose of this study was to examine the implementation of tools, whether these tools had been developed, determining the most appropriate approaches to implementation required author discussion and may have been subject to bias. Third, the small sample size we discovered during our scoping review carries a high risk of bias. Future reviews may need to have a greater focus on the gray literature to examine tools that have failed to be implemented in order to obtain a more cohesive picture of the state of digital surgical implementation in LMICs. This is because there may be a positive reporting bias with the already small number of published papers, as only successfully developed and implemented tools are being reported.

### Future Implications

Traditional methods of increasing surgical capacity in LMICs through mission trips have been criticized for lacking sustainability and for inadequate follow-up. As an alternative solution, systems that prioritize and conduct research on local sustainability and health system capacity have been proposed. Ideally, these systems would incorporate health care worker education and surgical training; therefore, it is the responsibility of the global surgeon to envision a new model that provides long-term educational support and knowledge [36]. Teaching must be a central and fundamental strategy in this regard; otherwise, the model of medical “voluntourism” will be implemented at the level of HIC institutions and forego involvement of LMIC institutions [36]. We advocate the use of digital surgical simulation for trainee education so that large foreign institutions can avoid this while continuing to play an important role in the education of surgeons from LMICs. AR and VR technologies are useful in the world of digital surgical simulation, but adaptation to the novel and long-term disruptions caused by the pandemic is required, and digital surgical simulations may play a crucial role in the training of surgeons in LMICs to increase surgical capacity [37]. The pandemic has unquestionably impacted the quality of access to traditional models of education through participation in or observation of surgical procedures. Lack of access to external training opportunities has exacerbated this problem, but digital surgical simulations provide a straightforward solution. We exercise caution when generalizing the effects of each implementation, as each region is unique and each innovation may require a different strategy in each community. Understanding the economic impacts of digital surgical simulation has been a crucial aspect of our paper, as this is one of the primary factors that may be considered crucial in the discussion of LMIC surgical trainees. Our findings demonstrate that despite the fact that many authors have made significant efforts to generate low-cost models, this often comes at the expense of fidelity, appropriateness, and sustainability of the tools—all of which COSECSA trainees rank as the most important aspects of their training [34]. This suggests that although authors may assume financial burdens are the most important factor, we propose that in fact the combination of all of these implementation factors is more than the sum of its parts, and we should avoid approaching aspects of development as the “most essential” components; rather we should develop a cohesive plan for implementation success. We urge innovators to work more closely with authors from LMICs to develop tools that can be built on top of existing technologies, as opposed to parachuting in novelties. Notwithstanding, we view these examples of innovation in LMICs as opportunities for reverse innovation, given that LMICs frequently have surgical populations presenting with more complex illness and provide a unique surgical approach that trainees in HICs may never encounter in their careers. Opportunities to engage in open radical hysterectomy, an approach largely replaced by laparoscopic approaches in HICs, is an illustration of this surgical approach [22]. However, the development of the VR toolkit for trainees in Zambia has created an intriguing opportunity to scale the learnings from LMICs to HICs [22].

### Conclusions

The scoping review on the implementation outcomes of digital surgical simulation implementation in LMICs revealed that participants, primarily medical students, and male residents rated surgical simulators and telecommunications devices highly for acceptability and usefulness, as they gained anatomical and procedural knowledge. However, image distortion, excessive light exposure, and video stream latency were commonly cited as shortcomings. The implementation cost varied by product, with the cost of development being a significant factor to consider. The study indicates that digital surgical simulation is a promising tool for medical education in LMICs, but further research is necessary to address some of the limitations and ensure successful implementation. In order to scale the use of these technologies in their respective environments, it is necessary to assign mentor-champions to promote their implementation. Acceptability and fidelity were rated quite highly in the majority of studies, and the reality of surgery and the virtual simulation were comparable; however, these are all technically feasible and there is a dearth of reporting on successful implementation. In addition, the use of low-cost 3D silicone models has been demonstrated, though they may not function as intended and require frequent updates and modifications. Therefore, it would be ideal to develop technology that is easily usable offline and pertinent to regional clinical practice. We urge more consistent reporting and understanding of implementation of science approaches in the development of digital surgical tools, as this is the critical factor that will determine whether we are able to meet the 2030 goals for surgical training in LMICs. Sustainability of implemented digital surgical tools are a pain point that must be focused on if we are to deliver digital surgical simulation tools to the populations that demand them the most. The limitations of the paper are that there was a high degree of inconsistency and vagueness in the reporting of implementation outcomes, a small sample size of papers, and a lack of inclusion of the gray literature. The suggested implication of our paper is to develop systems that prioritize local sustainability and health system capacity as opposed to traditional models of increasing surgical capacity in LMICs. We believe that digital surgical simulation can play a crucial role in training surgeons from these regions while allowing large foreign institutions to avoid implementation of unsustainable medical “voluntourism.”

## Appendix

Multimedia Appendix 1 PRISMA-ScR checklist of scoping review.

Multimedia Appendix 2 Comprehensive search strategy and extraction sheet.

## Abbreviations

AR: augmented reality
COSECSA: College of Surgeons of East, Central, and Southern Africa
HIC: high-income country
JBI: Joanna Briggs Institute
LMIC: low- and middle-income country
PRISMA-ScR: Preferred Reporting Items for Systematic Reviews and Meta-Analyses extension for Scoping Review
VR: virtual reality

## Data Availability

All data generated or analyzed during this study are included in this published article and its supplementary information files.
