# Supplementary material for: Current Implementation Outcomes of Digital Surgical Simulation in Low- and Middle-Income Countries: Scoping Review
Source: JMIR Med Educ. 2023 Jun 15;9:e23287. doi: 10.2196/23287 (PMC10337383; doi:10.2196/23287)
Supplement: Multimedia Appendix 2 [file mededu_v9i1e23287_app2.docx]

**APPENDIX 1**

***Comprehensive Search String***

*(Surg*) AND*

*(simulation OR digital OR virtual OR “virtual reality” OR augment* OR technology OR software OR simulator OR prototype* OR “oculus rift” OR robotics or haptics) AND*

*(training OR education OR learning) AND (sustainability OR feasibility or acceptability OR cost-effective* OR fidelity OR penetration OR implementation cost OR adoption OR appropriateness) AND*

*(rural OR lmic OR low-income countr*)*

**APPENDIX 2: Extraction Sheet**

Table 1: Summary of studies obtained through scoping review pertaining to the use of digital surgical simulation in LMICs and its implementation. VR = Virtual Reality, LMIC = Low-Middle Income Country

| Author (Year). Location. *Title*. | Objectives | Study Type. Population. Sample Size | Study Methodology | Key Findings | Strengths and Limitations |
| --- | --- | --- | --- | --- | --- |
| **Alvarez-Lopez et al. (2020),** Brazil, Spain, and Colombia  *Use of a Low-Cost Portable 3D Virtual Reality Gesture-Mediated Simulator for Training and Learning Basic Psychomotor Skills in Minimally Invasive Surgery: Development and Content Validity Study (x).* | To evaluate the fidelity of a newly developed web-based 3D VR simulator mediated by a gesture interface device for learning basic psychomotor skills for minimally invasive surgery. | Observational: Cross-sectional study  Survey Data   - Participant’s ratings on the fidelity of the gesture-based simulator   n=30   - Average age 42 years - 16 male, 14 female - 21 practicing surgeons (performed >100 minimally invasive procedures), 8 surgical residents (performed <100 MIS procedures), 1 non-medical | Participants were trained on the use of a newly developed MIS simulator and then performed 6 different surgical tasks multiple times within the simulator.  The computer collected data on their performance by tracking numbers of errors made and time spent to complete tasks.  Afterwards, participants completed a survey on the fidelity to criterion of the MIS simulator.  Participant’s survey opinions were scaled by a Likert scale. | 93% of participants rated the ability to realistically test hand-eye coordination as 4-5 and 87% gave this score for realism of depth perception.  100% of those surveyed believed that the prototype could be a solution for ubiquitous learning in minimally invasive surgery(rating it 4 or 5).  73% and 27% of participants assigned a rating of 5 and 4 respectively for ease of use.  For how well the physical forceps was represented in the virtual environment 73% rated 4-5.  97% of respondents considered this simulator enables learning of basic psychomotor skills in MIS with ratings of 4 or 5.  93% agreed that the tasks reflect the basic steps of a minimally invasive procedure, with ratings of 4 or 5.  The MIS simulator cost approximately $200 USD to construct. | Strengths:   - Demographics collection surveyed experience with video games, experience with minimally invasive surgical simulators, operating level, and level of training to account for confounding variables due to familiarity   Limitations:   - The simulator sacrificed minimally invasive surgery ergonomic principles to provide portability, lessing realism - Sample size was limited by availability. - The “cutting” skill was underrepresented in the tasks performed due to technical limitations of the simulator. |
| **Bing et al. (2021),** Zambia  *User Experience With Low-Cost Virtual Reality Cancer Surgery Simulation in an African Setting* | To evaluate gynecologic oncology trainee learning and user experience using a low-cost VR simulator to learn to perform an open radical abdominal hysterectomy in a resource-constrained setting. | Observational: Cross-sectional study  Survey Data   - Interview of participant’s experience with the simulator   n=11   - Average age 35.8 years - 6 male, 5 female - 8 senior medicine students, 2 resident trainees in obstetrics and gynecology, 1 fellow in gynecologic oncology | Participants received an in-person lecture on performing the radical abdominal hysterectomy and using VR for radical abdominal hysterectomy procedures.  They then were encouraged to use the simulator as frequently as they could within the allotted session time.  After each session, participants completed a self-evaluation, and then were interviewed in a semi-structured interview format. | - No participants interviewed described aspects of the simulation that felt incongruous with an actual surgical theater in Lusaka. - A goal was set of achieving 85% proficiency or higher in the simulator three times in a row. Participants noted that having a standard to strive for encouraged them to improve their skills further than they would have otherwise. - The participants interviewed expressed that the simulator increased anatomical and procedural knowledge. - Participants believed skills gained in the simulator transferred to other aspects of medical care and practice. | Strengths:   - Using a semi-structured interview format allowed for follow-up to structured questions and generated detailed qualitative information about the participant’s lived experiences.   Limitations:   - Participants noted they were motivated to keep using the simulator because it was a novel and rare mentorship opportunity - The medical students were interviewed as a group, which could influence their answers - Only six of the eleven participants were available for interview after using the simulator |
| **Bing et al. (2019),** Zambia  *Using Low-Cost Virtual Reality Simulation to Build Surgical Capacity for Cervical Cancer Treatment (x).* | To determine if a VR simulator using inexpensive computer gaming equipment could train surgery residents in a low-resource setting to perform a virtual open radical abdominal hysterectomy. | Observational: Cross-sectional Study  Study Data   - Movement efficiency and time efficiency scores for each user after each simulation run   n=10   - Average age 35.8 years - 6 male, 4 female - 8 Senior medical students - 1 obstetrics and gynecology resident and 1 gynecologic oncology fellow | Participants were interviewed on demographics and taught to use the VR radical abdominal hysterectomy simulator. Participants were encouraged to use the simulator as frequently as they could within the allotted session times and until they reached a proficiency score of 85 or higher five times in a row. Their scores of procedural, movement, and time efficiency were collected and analyzed. | Movement efficiency and time efficiency of the simulation was strongly associated with the number of simulations performed (p<0.001) and level of surgical experience (p<0.001).  Younger participants were more time efficient than older participants (p<0.001). | Strengths:   - Broad demographic collection to account allow multiple interpretations of the VR simulator score results - Included a complex self-assessment form that allowed participants to identify substeps needing improvement, strengthening motivation   Limitations:   - A small sample size limited by availability - Only tested one surgical procedure |
| **Bolton et al. (2021),** Sierra Leone  *The vital study: a feasibility study of a randomised controlled trial to examine if virtual reality technology can improve surgical training in sierra leone* | To determine the feasibility of implementing a VR module in Sierra Leone and if the technology improves learning and skill acquisition during training. | Randomized control trial and Survey  Study Data   - Standardized test scores were collected after the control and intervention groups completed training courses - A survey collecting both groups feasibility ratings of their courses   n=30. 15 VR intervention, 15 control   - Average age 32.3 years - 24 male, 6 female - All participants were general surgery residents | Participants were randomly divided into two groups. The intervention group watched a VR simulation of lower limb amputation surgical training that was viewed before a course on lower limb amputation (control group did not watch the VR pre-course material).  After the course ended, both groups of participants were evaluated by formative assessments and then asked to complete a survey on the course’s feasibility. | The VR instructed group performed on average higher scores in formative assessments, however this result was not statistically significant.  The VR instructed group rated a statistically significant higher rating of engagement in the course. The VR group also noted increased perceived learning over the control group (p<0.05). | Strengths:   - Accounted for many participant demographics in analysis of results such as specialty, smartphone use, and previous surgical training. - Study used both quantitative scoring as well as self-reporting   Limitations:   - Lack of generalizability to other surgical procedures. - Lack of statistically significant quantitative figures. - How VR was used not directly stated - could be to watch a video or to interact. |
| **Bunogerane et al. (2018),** Rwanda  *Using Touch Surgery to Improve Surgical Education in Low- and Middle-Income Settings: A Randomized Control Trial.* | To determine the effectiveness of training with Touch Surgery and its feasibility as a surgery education tool in a low-resource setting. | Randomized control trial and Survey  Study Data   - Participant’s tendon repair operative skills and theory measured practically and through MCQ. - A survey collecting participant’s feasibility ratings of the technology   n=27 14 Touch Surgery users, 13 control   - 25 male, 2 female - All participants were University of Rwanda surgery students with resident specialties below   - 14 general surgery   - 11 orthopedics   - 2 urology | Participants completed a pre-simulation demographic and confounder questionnaire. Participants completed a MCQ tendon repair test. Participants were then randomized to study with Touch Surgery or with a textbook, both had comparable information on tendon repair technique. Participants were allowed as much time as they wanted before performing the procedure with a real tendon. Independent expert raters rated the simulation with a standardized rubric. Participants repeated the MCQ test, and then a post simulation survey. | - 92.3% of the assigned Touch Surgery participants reported it presented a useful or very useful training and assessment tool - 84.6% reported it as useful or very useful for rehearsal before an operation - 76.9% reported it as useful or very useful to learn more procedures - 84.6% reported it as useful or very useful in a surgical training program - Operative skills measured by tendon repair simulation overall rubric score was 89.71% for Touch Surgery users and 63.4% for textbook learning users (p<0.0001). - Tendon repair theory MCQ test scores were improved 13% by the textbook and 39.1% for the TS group (p=0.535 and 0.056, respectively) - Tendon repair technique MCQ test scores were improved 19% for textbook learners and 38.1% for Touch Surgery group (p=0.165 and 0.0254, respectively) | Strengths:   - Inter-rater reliability was accounted for with checking scoring of other rater - Confounding variables were accounted for in the demographic questionnaire   Limitations   - The study collected participants by volunteer |
| **McCullough et al. (2018),** Mozambique  *Google Glass for Remote Surgical Tele-proctoring in Low- and Middle-income Countries: A Feasibility Study from Mozambique.* | To demonstrate the effectiveness of wearable technology real-time tele-proctoring for connecting surgical providers in LMIC to professionals in high-resource settings. | Case study: Outcome evaluation  Survey Data   - A log recorded all procedures performed with the GG technology as well as preoperative screenings and postoperative evaluations - Notes were taken on any interruptions in the stream - Notes were taken on complications experienced by the patient intraoperatively and postoperatively   n=2   - Two male surgeons - The field surgeon positioned in Mozambique - The mentor plastic surgeon positioned in USA - 12 surgical procedures performed and recorded from Aug 2017 - Feb 2018 | A field surgeon positioned in Mozambique was connected to a mentor surgeon in the USA through this tele-mentoring GG setup. 12 surgeries were performed and recorded in Mozambique by the field surgeon with the GG. All surgeries were live-interacted with the mentor surgeon over the internet through computers. The mentor surgeon was able to observe and assist the field surgeon verbally through their tele-mentoring setup while he operated. Cases selected for the study were selected on two criteria: (1) had to be representative of common presentations encountered by the field surgeon and (2) utilize reconstructive approaches that would be novel to him. Notes were taken by both surgeons on their experience with the technology after each use. | - Both participants reported moderate visual impairment due to image distortion from motion and light overexposure. - Video stream latency and connection disruptions were also cited as limitations. - Both participants reported that the technology was highly useful as a training tool in both the intraoperative and perioperative settings. - The participants rated that the degree of impairment was significant and moderate due to poor / inconsistent reproduction of detail. - The ExpertEye software used to process the Google Glass feed has an expensive ($6990USD/year) subscription fee | Strengths:   - Common cases were used to replicate real-life scenarios - Multiple surgeries were performed to allow for more errors to occur   Limitations:   - The field surgeon only performed surgeries in the same hospital in Mozambique - Google Glass was prone to parallax |
| **Rosser et al. (1999),** Ecuador  *Use of mobile low-bandwith telemedical techniques for extreme telemedicine applications* | To gauge the effectiveness and feasibility of a novel telementoring application in an underdeveloped part of the world. | Case study: Outcome evaluation  Study Data   - Patient interview after surgery - Recording of the telementoring session’s stability   n=5   - 5 patients pre-operative evaluation on by a remote surgery team | A mobile operating room in a remote region of Ecuador used a laptop computer equipped with telemedicine software, a videoconferencing system, and a digital camera. Surgical patients were evaluated and operative decisions were made over a low-bandwidth telephone line (12kbps). Surgeons in the mobile operating room were telementored by an experienced surgeon located at Yale University. | - One disconnection per patient interview session occurred. - Physicians at Yale University were able to witness and tele-mentor the mobile operating room physicians in real time. | Strengths:   - Internet speed was constantly monitored   Limitations:   - Limited sample size of only 5 operations - Mobile station never recorded at another site |
